# A new deletion allele of *sma-4*

**DOI:** 10.17912/z4z9-ce10

**Published:** 2018-11-09

**Authors:** Alexandra N McKillop, Herong Shi, Jun Liu

**Affiliations:** 1 Department of Molecular Biology and Genetics, Cornell University, Ithaca, NY 14853

**Figure:**
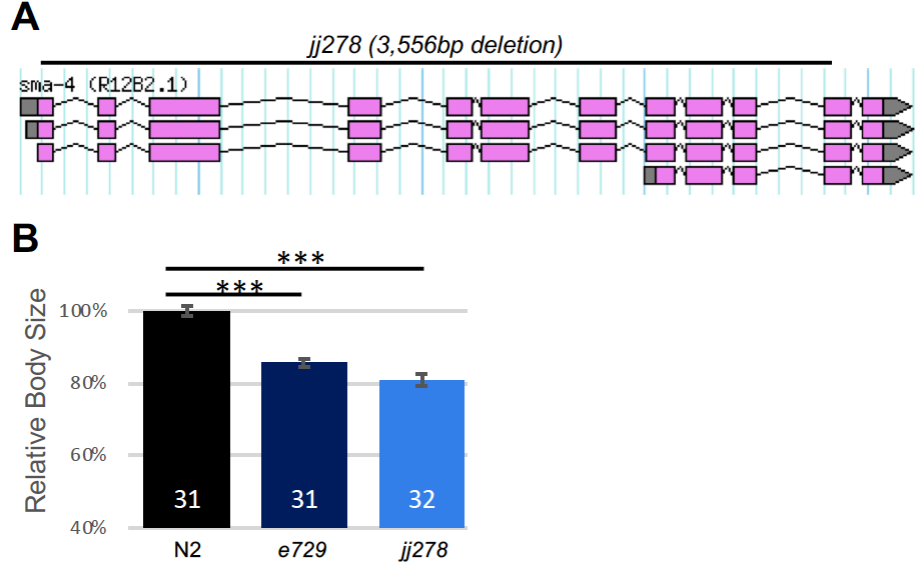
**1A.** Location of the *jj278* deletion. **1B.** Results of body size measurement. *jj278* worms are small. *e729* is the canonical allele of *sma-4* and was used as a control. Body size measurements were conducted as previously described (TIAN *et al.* 2013). Hermaphrodite worms were imaged at the L4.1 stage based on vulva development (MOK *et al.* 2105), Body lengths were measured from the images using the Segmented Line tool of Fiji. Statistical analysis was carried out using Student’s *t* test. The mean body length of N2 worms is normalized to 100%. Error bars represent 95% confidence intervals for the normalized body length.­ The numbers inside each bar represent the numbers of animals measured for the specific genotype. *** *P*<0.0001.

## Description

*sma-4* encodes the co-Smad of the BMP pathway in *C. elegans*, which is also known as the Sma/Mab pathway (Savage *et al.* 1996). Null mutations in core components of this pathway, including the BMP ligand DBL-1, the receptors SMA-6 and DAF-4, and the R-Smads SMA-2 and SMA-3, all result in a small body size phenotype without significantly compromising viability (Gumienny and Savage-Dunn 2013). However, we found that even after multiple rounds of out-crossing, two deletion alleles of *sma-4, ok3140* and *tm4731*, caused late larval lethality and embryonic lethality, respectively. This observation suggests that either SMA-4 has DBL-1/BMP-independent functions that are required for viability, or *ok3140* and *tm4731* have closely linked lethal mutations. To distinguish between these two possibilities, we generated a deletion allele of *sma-4* using CRISPR/Cas9-mediated non-homologous end joining. This allele, *jj278*, contains a 3,556bp deletion (position: Chromosome III: 5,816,203….5,819,759) that deletes almost the entire coding region of *sma-4* (Figure 1A) and represents a true molecular null. *jj278* animals are viable and fertile, but are smaller than wild-type animals (Figure 1B), like loss-of-function mutants in other core BMP pathway members (Savage *et al.* 1996; Krishna *et al.* 1999). This result suggests that *ok3140* and *tm4731* likely have closely linked lethal mutations not caused by their respective *sma-4* deletions.

## Reagents

**Plasmids and oligos used to generate *jj278*:**

All sgRNA plasmids were using the pRB1017 backbone (ARRIBERE et al. 2014).

pJKL1171 (*sma-4* N-sgRNA #1):

JKL-1692 (F): TCTTGGTCGAATAATGTTTCATCC

JKL-1693 (R): AAACGGATGAAACATTATTCGAC

pJKL1172(*sma-4* N-sgRNA #2):

JKL-1694 (F): TCTTGACGGCTGAGATGTCATACC

JKL-1695 (R): AAACGGTATGACATCTCAGCCGT

pANM1 (*sma-4* C-sgRNA #1):

ANM-12 (F): TCTTGCACTGTCAGGCATTATCGC

ANM-13 (R): AAACGCGATAATGCCTGACAGTGC

pANM2 (*sma-4* C-sgRNA #2):

ANM-10 (F): TCTTGCAGCGATAATGCCTGACAG

ANM-11 (R): AAACCTGTCAGGCATTATCGCTGC

Oligos used to genotype *jj278*:

Expected sizes: Wild-type: 4.097kb + 295bp; *jj278*: 541bp

JKL-1104: CATGAATATGAGAAACTGCTGG

ANM-14: CCGTTGTCACCTCGAATCAC

ANM-15: GCTCTGCTCCATACAAAGGATC

**Strain:** LW5558: *sma-4(jj278)* III. Will be sent to the CGC.
